# The Brown Alga *Stypopodium zonale* (Dictyotaceae): A Potential Source of Anti-*Leishmania* Drugs

**DOI:** 10.3390/md14090163

**Published:** 2016-09-08

**Authors:** Deivid Costa Soares, Marcella Macedo Szlachta, Valéria Laneuville Teixeira, Angelica Ribeiro Soares, Elvira Maria Saraiva

**Affiliations:** 1 Instituto de Microbiologia Paulo de Góes, Universidade Federal do Rio de Janeiro, Rio de Janeiro 21.941-902, Brazil; soaresdc@micro.ufrj.br (D.C.S.); marcellaszlachta@hotmail.com (M.M.S.); 2 Laboratório Produtos Naturais de Algas Marinhas (ALGAMAR), Departamento de Biologia Marinha, Instituto de Biologia, Universidade Federal Fluminense, Niterói 24.210-150, Brazil; valerialaneuville@gmail.com; 3 Grupo de Produtos Naturais de Organismos Aquáticos (GPNOA), Núcleo em Ecologia e Desenvolvimento Sócioambiental de Macaé (NUPEM), Universidade Federal do Rio de Janeiro, Campus-Macaé, Rio de Janeiro 27.965-045, Brazil; angelica.r.soares@gmail.com

**Keywords:** marine natural products, meroditerpenes, *Stypopodium zonale*, leshmanicidal activity

## Abstract

This study evaluated the anti-*Leishmania amazonensis* activity of a lipophilic extract from the brown alga *Stypopodium zonale* and atomaric acid, its major compound. Our initial results revealed high inhibitory activity for intracellular amastigotes in a dose-dependent manner and an IC_50_ of 0.27 μg/mL. Due to its high anti-*Leishmania* activity and low toxicity toward host cells, we fractionated the lipophilic extract. A major meroditerpene in this extract, atomaric acid, and its methyl ester derivative, which was obtained by a methylation procedure, were identified by nuclear magnetic resonance (NMR) spectroscopy. Both compounds inhibited intracellular amastigotes, with IC_50_ values of 20.2 μM (9 μg/mL) and 22.9 μM (10 μg/mL), and selectivity indexes of 8.4 μM and 11.5 μM. The leishmanicidal activity of both meroditerpenes was independent of nitric oxide (NO) production, but the generation of reactive oxygen species (ROS) may be at least partially responsible for the amastigote killing. Our results suggest that the lipophilic extract of *S. zonale* may represent an important source of compounds for the development of anti-*Leishmania* drugs.

## 1. Introduction

Leishmaniasis comprises a wide spectrum of diseases that are characterized by cutaneous, mucosal, and visceral organ lesions. The form and morbidity of the disease are dependent upon both the *Leishmania* species and immunological status of the host [1]. Leishmaniasis affects all continents and approximately 0.2 to 0.4 million cases of visceral leishmaniasis and 0.9 to 1.2 million cases of cutaneous leishmaniasis occur annually, causing significant morbidity and mortality. Thus, leishmaniasis is recognized as one of the most neglected tropical diseases for which drug development has been stimulated by the Drugs for Neglected Diseases Initiative [2]. Currently, pentavalent antimonials, pentamidine, amphotericin B, and paromomycin are the drugs available for the treatment of leishmaniasis. However, all of these drugs exhibit toxicity, adverse side effects, and increased incidence of the emergence of drug-resistant strains, which reinforces the need to develop new approaches for leishmaniasis therapy [2,3].

Marine organisms have been studied as an important source of biologically-active secondary metabolites [4,5]. However, few studies have assessed the leishmanicidal activity of algae extracts [6,7,8,9,10,11,12,13,14,15,16]. The brown algae of genus *Stypopodium* (Dictyotaceae) is widespread in both tropical and subtropical regions, and has been well recognized as a rich source of structurally-unique and biologically-active diterpenes of mixed biogenesis (meroditerpenoids) [16,17,18,19,20,21,22,23]. These compounds exhibit interesting pharmacological activities, such as antitumoral [24], insecticidal [25], and antiviral [23,26] effects, and also plays an ecological role by providing chemical defense against herbivory [27].

Here, we describe the anti-leishmanial activity of lipophilic extract of *Stypopodium zonale* and meroditerpenoid atomaric acid, the major compound isolated from the lipophilic extract of *S. zonale*. Additionally, the methyl ester derivative of atomaric acid was obtained by a methylation procedure and tested against the same parasites. The extract of the compounds, atomaric acid, and its methyl ester derivative, inhibited the growth of *Leishmania amazonensis* intracellular amastigotes in infected macrophages and exhibited low toxicity for the host cells. These findings characterize *Stypopodium zonale* as a potential source of substances for the development of drugs for leishmaniasis treatment.

## 2. Results

### 2.1. Crude Extract Analysis and Structural Elucidation of Pure Compounds

**Figure 1:**
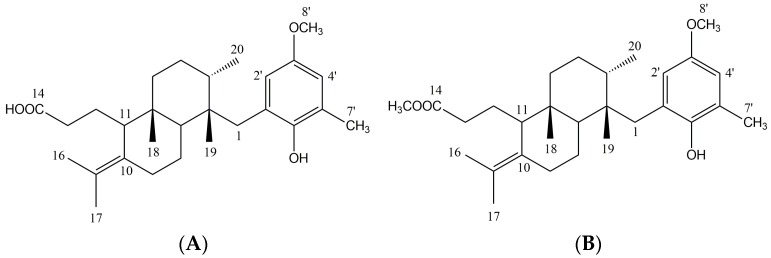
Chemical structure of (**A**) Atomaric acid (ATA) and (**B**) its methyl ester derivative (AAE).

Specimens of *Stypopodium zonale* (J.V. Lamouroux) Papenfuss were collected in Búzios, Rio de Janeiro State, Brazil. The dichloromethanic extract of *Stypopodium zonale* (SZE) was analyzed by both 1D and 2D nuclear magnetic resonance (NMR) spectroscopy. Characteristic signals for meroditerpenoids were observed for the major compounds. SZE was fractionated by SiO_2_ chromatography to yield atomaric acid (ATA), identified as the major compound in the extract. This known meroditerpenoid and its methyl ester derivative AAE (Figure 1) obtained by a methylation procedure were identified by spectroscopy in comparison with previously reported data [17,26].

*Atomaric acid* (ATA): ^1^H-NMR (CDCl_3_, 300 MHz) δ: 0.93 (s, 3H, H-19), 1.02 (s, 3H, H-18), 1.15 (d, 3H, *J* = 6.9 Hz, H-20), 1.26 (d, 1H, *J* = 14.4, H-4a), 1.38 (dd, 1H, 6.0 e 12.0, H-7), 1.49 (m, 2H, H-5), 1.51 (m, 1H, H-8b), 1.57 (m, 1H, H-12a), 1.66 (s, 3H, H-17), 1.68 (s, 3H, H-16), 1.73 (m, 1H, H-3), 1.74 (m, 1H, H-8a), 1.81 (m, 1H, H-12b), 1.88 (m, 1H, H-4b), 1.96 (m, 1H, H-9a), 2.22 (s, 3H, H-7′), 2.25 (d, 1H, *J* = 13.8, H1a), 2.26 (m, 2H, H-13), 2.32 (m, 1H, H-11), 2.39 (m, 1H, H-9b), 2.84 (d, 1H, *J* = 13.8, H-1b), 3.73 (s, 3H, 8′–OCH_3_), 6.54 (d, 1H, *J* = 3.00 Hz, H-4′), 6.69 (d, 1H, *J* = 3.00 Hz, H-2’). ^13^C-NMR (CDCl_3_) *δ*: 15.5 (C-20), 17.5 (C-7′), 17.6 (C-18), 20.4 (C-16, C-19), 20.6 (C-17), 22.2 (C-8), 23.3 (C-9), 24.9 (C-4, C-12), 34.7 (C-1), 35.0 (C-3), 32.7 (C-13), 36.1 (C-5), 38.5 (C-6), 40.0 (C-2), 41.7 (C-7), 52.6 (C-11), 55.0 (8′-OCH_3_), 112.8 (C-4’), 113.5 (C-2′), 122.2 (C-10), 125.6 (C-6′), 128.2 (C-3′), 133.3 (C-15), 147.5 (C-1′), 151.7 (C-5′), 174.8 (C-14).

*Methyl ester* of the *Atomaric acid* (AAE): ^1^H-NMR (CDCl_3_, 300 MHz) δ: 0.93 (s, 3H, H-19), 1.02 (s, 3H, H-18), 1.15 (d, 3H, *J* = 8.0 Hz, H-20), 1.26 (m, 1H, H-4a), 1.38 (m, 1H, H-7), 1.49 (m, 2H, H-5), 1.51 (m, 1H, H-8b), 1.57 (m, 1H, H-12a), 1.66 (s, 3H, H-17), 1.68 (s, 3H, H-16), 1.73 (m, 1H, H-3), 1.74 (m, 1H, H-8a), 1.81 (m, 1H, H-12b), 1.88 (m, 1H, H-4b), 1.96 (m, 1H, H-9a), 2.22 (s, 3H, H-7′), 2.26 (m, 2H, H-13), 2.32 (m, 1H, H-11), 2.39 (m, 1H, H-9b), 2.41 (d, 1H, *J* = 14.0, H1a), 2.84 (d, 1H, *J* = 14.0, H-1b), 3.72 (s, 3H, 8′–OCH_3_), 3.65 (s, 3H, –COOCH_3_), 4.27 (sl, –OH), 6.54 (d, 1H, *J* = 3.00 Hz, H4′), 6.69 (d, 1H, *J* = 3.00 Hz, H-2′). ^13^C-NMR (CDCl_3_) δ: 15.7 (C-20), 16.8 (C-18), 17.9 (C-7′), 20.4 (C-16, C-19), 20.7 (C-17), 22.2 (C-8), 23.3 (C-9), 25.0 (C-4, C-12), 33.0 (C-13), 35.6 (C-1, C-3), 36.6 (C-5), 38.9 (C-6), 40.6 (C-2), 41.9 (C-7), 51.4 (–COOCH_3_), 53.1 (C-11), 55.5 (8′–OCH_3_), 113.2 (C-4′), 114.7 (C-2′), 123.2 (C-10), 124.0 (C-6′), 126.8 (C-3′), 133.0 (C-15), 146.8 (C-1′), 152.6 (C-5′), 174.7 (C-14).

### 2.2. Anti-Leishmania Activity of Stypopodium zonale Extract

**Figure 2:**
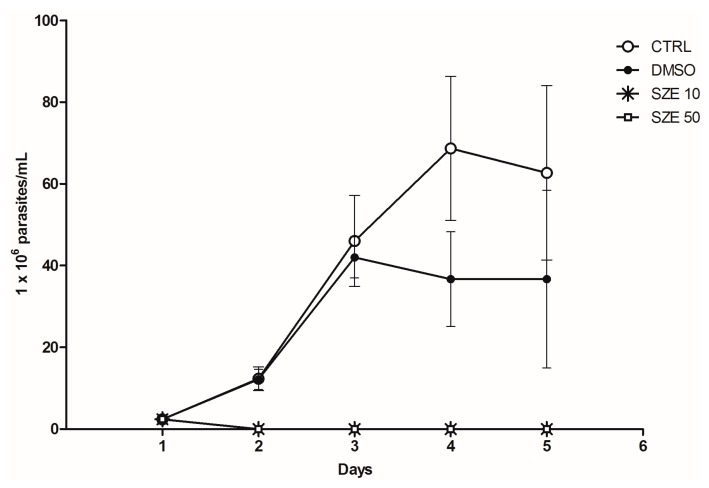
Effects of SZE against *Leishmania amazonensis* promastigotes. Promastigotes were treated once with different concentrations of SZE (open squares and asterisks). 0.05% dimethyl sulfoxide (DMSO) (SZE diluent; black circle) and parasites maintained in medium were used as controls (open circle). Anti-promastigote activity was estimated by counting viable parasites over a period of four days. The results from three experiments are shown as parasite numbers ± SEM.

**Figure 3:**
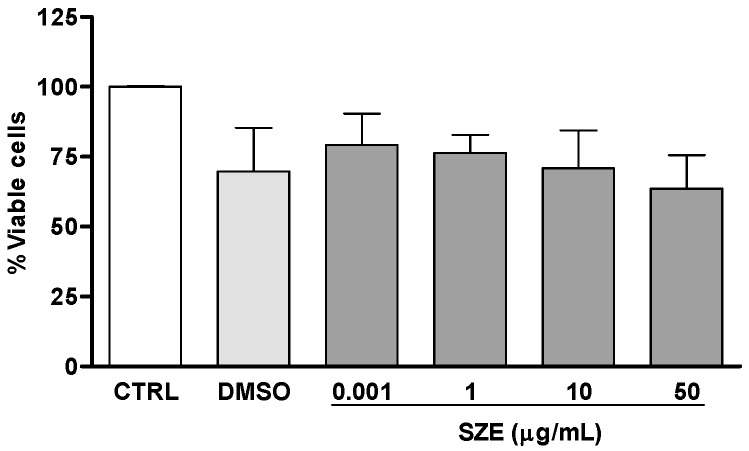
Safety of SZE against macrophages. Peritoneal macrophages were incubated for 24 h with the indicated concentrations of SZE or 1% DMSO (vehicle); cell viability was assessed by the XTT assay. Data from three independent experiments carried out in triplicate are expressed as % viable cells compared with controls.

**Figure 4:**
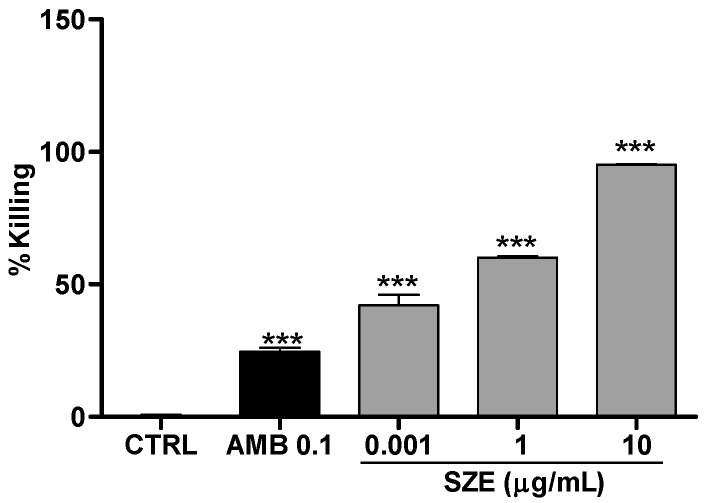
Anti-amastigote activity of SZE. Peritoneal murine macrophages infected with *Leishmania amazonensis* were treated with SZE at the indicated concentrations. DMSO at 0.01% (vehicle) and Amphotericin B [AMB] at 0.1 μM were used as controls. Amastigote growth was assessed 24 h after SZE treatment. The results from three experiments performed in duplicate are shown as the percentage of amastigotes killing ± SEM compared with an untreated control (CTRL); *** *p* < 0.0001 compared with controls.

Initially, we investigated the potential leishmanicidal effects of SZE on *Leishmania amazonensis*. Thus, promastigotes were treated with 10 or 50 μg/mL of SZE and parasite viability was evaluated. Our findings showed that SZE inhibited 100% of promastigote growth two days after treatment at both concentrations that we assayed (Figure 2). To test the safety of SZE in mammalian cells, macrophages were treated with different concentrations of SZE and cell viability was assessed using the 2,3-bis(2-methoxy-4-nitro-5-sulfophenyl)-2*H*-tetrazolium-5-carboxanilide inner salt (XTT) assay. Treatment with SZE up to a concentration of 50 μg/mL was not toxic for host cells (Figure 3). The anti-amastigote activity of SZE was evaluated in *L. amazonensis*-infected peritoneal macrophages treated for 24 h with different concentrations of SZE. Our results indicated that SZE inhibited intracellular amastigotes in a concentration-dependent manner, with 42%, 60% and 95.2% inhibition at 0.001, 1, and 10 μg/mL, respectively, while amphotericin B (AMB) at 0.1 μg/mL caused a 24.5% reduction in amastigote growth (Figure 4). The IC_50_ of SZE for amastigotes was 0.27 μg/mL.

### 2.3. Leishmanicidal Activity of Atomaric Acid (ATA) and Its Methyl Ester Derivative (AAE)

**Figure 5:**
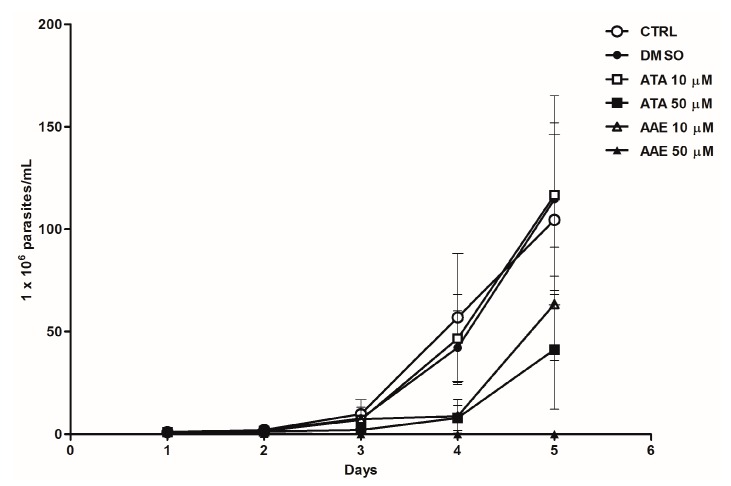
Effects of ATA and AAE against *Leishmania amazonensis* promastigotes. Promastigotes were treated once with ATA and AAE, and parasite viability was quantified daily. ATA 10 μM (open square) and 50 μM (black square); AAE 10 μM (open triangle) and 50 μM (black triangle); control (open circle); and DMSO vehicle (black circle). The results from two experiments are shown as parasite numbers ± SEM.

To identify the anti-leishmanial active compounds in SZE, we first tested ATA, the major compound present in this extract, along with its derivative AAE on the proliferation of *L. amazonensis* (Figure 5). ATA and AAE at 50 μM inhibited promastigote growth by up to 86% and 100%, respectively, after three days with only a single treatment.

**Figure 6:**
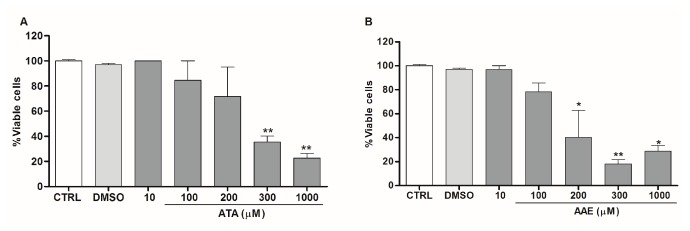
Safety of ATA and AAE against macrophages. Peritoneal macrophages were incubated for 24 h with the indicated concentrations of ATA (**A**), AAE (**B**), or 1% DMSO (vehicle); cell viability was assessed by the XTT assay. Data from two independent experiments carried out in triplicate are expressed as % viable cells compared with controls; * *p* < 0.05, ** *p* < 0.01 compared with controls.

To test the safety of ATA and AAE on host cells, we evaluated the dehydrogenase activity of macrophages using the XTT method. We found that treatment of macrophages with ATA or AAE only affected dehydrogenase activities at high concentrations (Figure 6A,B). ATA was cytotoxic for macrophages at 300 μM, affecting 64.5% of cells viability, while AAE at 200 μM affected the viability of 60% of macrophages. The CC_50_ values for macrophages treated with ATA and AAE were 169.5 μM (75 μg/mL) and 262.5 μM (209 μg/mL), respectively (Table 1).

**Figure 7:**
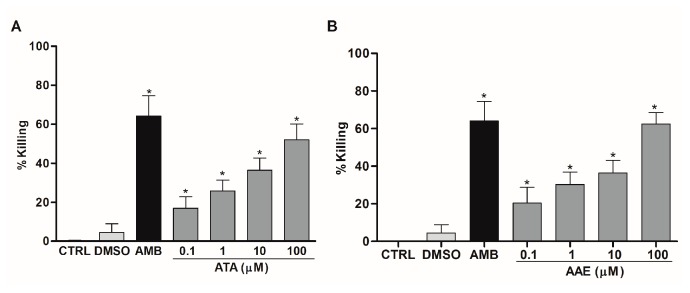
Activity of ATA and AAE against intracellular amastigotes. Peritoneal murine macrophages infected with *Leishmania amazonensis* were treated with ATA (**A**) or AAE (**B**) at the indicated concentrations. DMSO at 0.01% (Vehicle) and Amphotericin B (AMB) at 1 μM were used as controls. Amastigote growth was assessed 24 h post-SZE treatment. The results are from three and two experiments performed in duplicate for ATA and AAE, respectively, are shown as % of amastigotes killing ± SEM compared with untreated controls (CTRL); * *p* < 0.05 compared with controls.

To evaluate the anti-amastigote activity of ATA and AAE, we treated infected macrophages with these compounds. We found that a 24 h treatment with ATA at 0.1, 1, 10, and 100 μM killed 17%, 26%, 36%, and 52% of amastigotes, respectively (Figure 7A). AAE administered at the same concentrations killed 20%, 30%, 36%, and 62% of the amastigotes (Figure 7B). The IC_50_ values of ATA and AAE were 20 μM (9 μg/mL) and 23 μM (10 μg/mL), respectively (Table 1).

**Figure 8:**
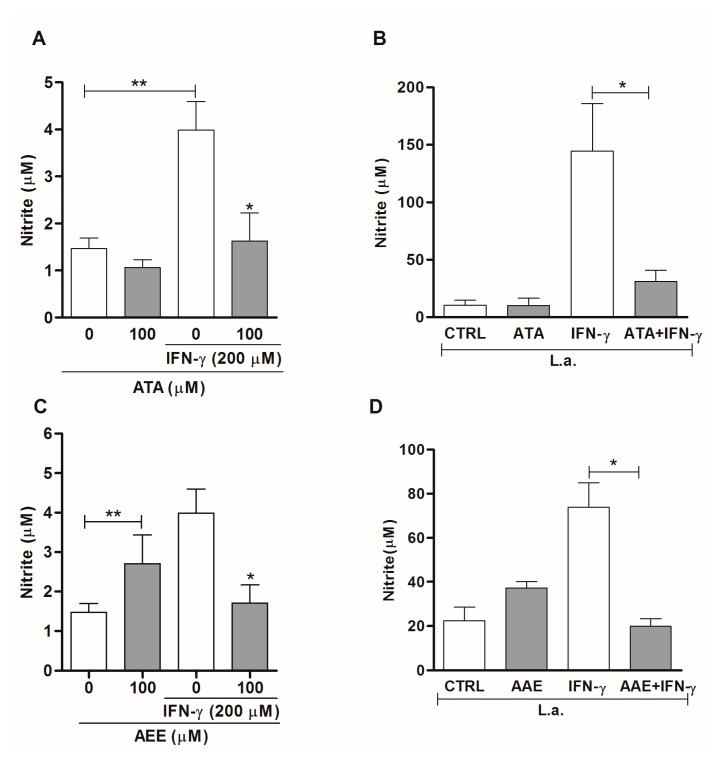
Nitric oxide (NO) production by macrophages is affected by ATA and AAE. Uninfected macrophages (**A**,**C**) and *Leishmania* infected-macrophages (**B**,**D**) at a 10:1 ratio were stimulated or not with 200 μM IFN-γ and were incubated in the presence or absence of either 100 μM ATA (**A**,**B**) or 100 μM AAE (**C**,**D**). NO production was evaluated after 48 h of treatment using the Griess method. The results from three independent experiments that were performed in duplicate are shown as the mean nitrite concentrations ± SEM; ** *p* < 0.01; * *p* < 0.05.

As nitric oxide (NO) and reactive oxygen species (ROS) are potent leishmanicidal mediators, we studied whether ATA and AAE could modulate these effector molecules in macrophages to kill intracellular amastigotes. Treatment with ATA or AAE of uninfected macrophages that were stimulated or not with IFN-γ resulted in no significant changes in the production of NO (Figure 8A,C). By contrast, infected macrophages stimulated with IFN-γ exhibited a 75% and 73% reduction in NO production after treatment with ATA and AAE at 100 μM, respectively (Figure 8B,D).

**Figure 9:**
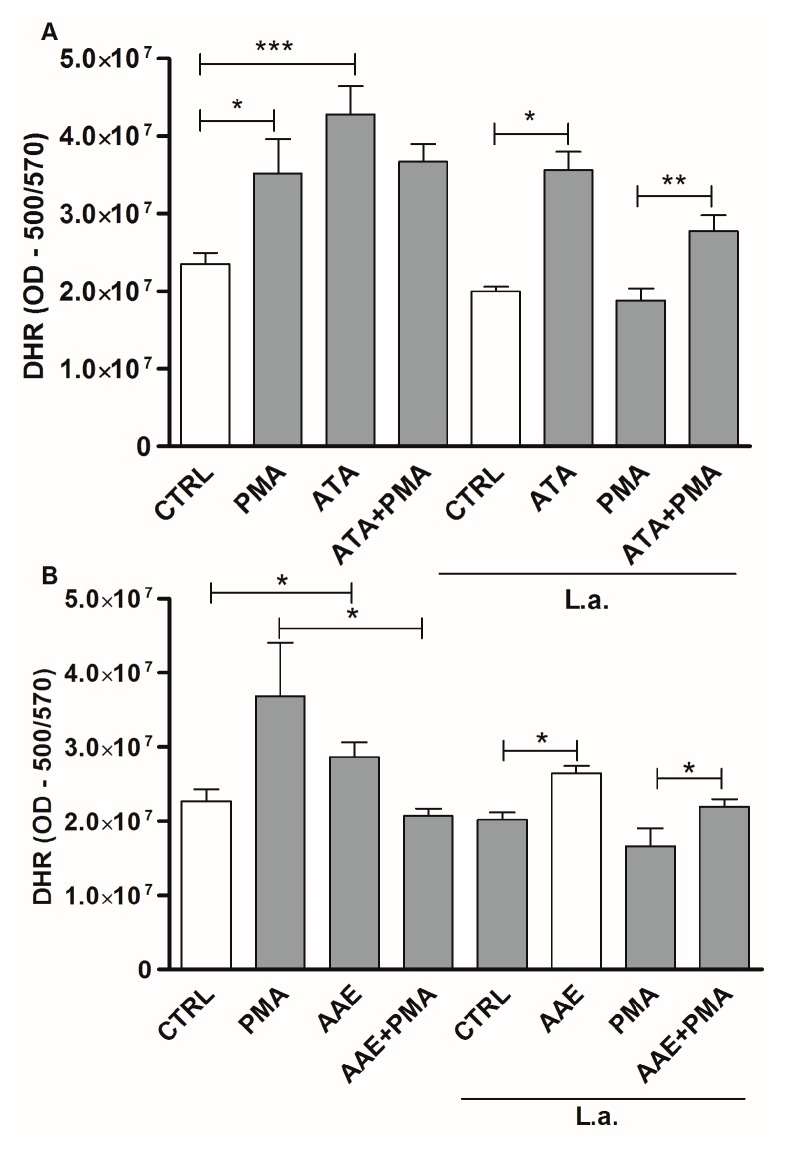
ROS production by macrophages is affected by ATA and AAE. Uninfected macrophages and macrophages infected with *Leishmania amazonensis* at a 10:1 ratio were stimulated or not with PMA and incubated in the presence or absence of 100 μM ATA (**A**) or 100 μM AAE (**B**) for 30 min. Macrophages were then stained with 50 μM dihydrorhodamine 123 (DHR). Data represent means ± SEM of three independent experiments performed in triplicate; *** *p* < 0.0001; ** *p* < 0.01; * *p* < 0.05.

By analyzing ROS production, we found that treatment of uninfected macrophages with 100 μM ATA increased ROS by 82% compared with untreated controls (Figure 9A). Similar results were observed in macrophages treated with phorbol 12-myristate13-acetate (PMA), a classical ROS inducer (Figure 9A). Evaluating infected macrophages, a similar increase was observed after treatment with 100 μM ATA compared with untreated controls, although infected macrophages were unable to respond to PMA stimulation (Figure 9A). We also observed modulation of ROS levels in macrophages treated with AAE. Thus, treatment with 100 μM AAE increased ROS production by 62% and 31% in infected and uninfected macrophages, respectively (Figure 9B). Both ATA and AAE reversed ROS inhibition induced by *Leishmania* after PMA treatment of infected macrophages, and increased ROS production 1.3-fold compared to infected macrophages treated with PMA alone (Figure 9A,B).

## 3. Discussion

In this present study, we showed a leishmanicidal activity of *Stypopodium zonale* extract (SZE) against *Leishmania amazonensis*, promastigotes, and intracellular amastigotes. SZE inhibited intracellular amastigotes growth in a time- and concentration-dependent manner.

Currently, anti-promastigote activity has been demonstrated for algae extracts from various other species, such as *Caulerpa sertularioides*, *Gracillaria corticata*, *Gracillaria salicornia*, and *Sargassum oligocystum*, which inhibited the growth of *L. major* promastigotes [28].

However, there are no previously published reports of algae extracts for activity against intracellular amastigotes, as only tests of axenic amastigotes have been reported. Our results showed that SZE could kill intracellular amastigotes with an IC_50_ value of 0.27 μg/mL. Studies comparing the leishmanicidal activity of 32 algae species against axenic amastigotes reported that only four of those exhibited an IC_50_ value below 20 μg/mL, which suggests that SZE is one of the most active extracts that has been previously studied [13,14,15]. These data added, along with the low toxicity of SZE for host cells, demonstrates its potential as a source of molecules for leishmanicidal drug development.

With an aim to identify the compounds responsible for the strong anti-leishmanial activity that we observed, SZE was fractionated and atomaric acid (ATA) was characterized as its major compound. This meroditerpene is a dominant compound in certain *Stypopodium zonale* populations [18], and it may be involved in important ecological interactions within the marine environment [27,28,29,30]. The biological activities of ATA have been previously reported [18,23,26], but its leishmanicidal activity was demonstrated for the first time in this present study. Herein, we also tested the methyl ester derivative of the atomaric acid (AAE), a semi-synthetic compound obtained by a usual chemical modification approach, to evaluate whether a less-polar version of ATA would show increased activity.

Similar to SZE, both ATA and AAE showed activity against the promastigotes and intracellular amastigotes of *L. amazonensis*, along with low toxicity for host cells. The anti-*Leishmania* activities of ATA and AAE were comparable, showing IC_50_ values for intracellular amastigotes of 20 μM (9 μg/mL) and 23 μM (10 μg/mL), and selectivity indexes (SI) of 8.4 (8.3) and 11.5 (21), respectively, suggesting that derivatization of ATA could improve its activity by reducing its CC_50_ by 2.8-fold. SZE was 33- and 37-fold more active than ATA and AAE, respectively. This difference likely resulted from the association of these major compounds with minor substances present in SZE, suggesting a possible synergism among these compounds.

The anti-leishmanial activity of substances isolated from algae has been previously reported for terpenes [14,16]. The halogenated sesquiterpenes, elatol and obtusol, which were isolated from the red alga *Laurencia dendroidea*, as well as the diterpene dolabelladienotriol obtained from the brown alga *Dictyota pfaffii* (Dictyotaceae), were active against the promastigote and amastigote forms of *L. amazonensis*. Here, our findings suggested an anti-leishmanial activity for two meroditerpenes, ATA, and its derivate AAE, establishing the robust leishmanicidal potential of algal terpenes.

We determined that ATA and AAE modulate macrophage activity by inhibiting NO production, which is an important mediator of *Leishmania* killing. ATA and AAE treatment reduced NO production in infected macrophages stimulated by IFN-γ. These data suggest that the *Leishmania* killing mediated by ATA and AAE occurred independently of NO production. Similarly, dolabelladienotriol, an algae-isolated substance with anti-amastigote activity, can also inhibit NO production [16]. In contrast to ATA and AAE, dolabelladienotriol can inhibit NO in both infected and uninfected macrophages that are stimulated or not with IFN-γ + LPS [16]. Recently, Kar and colleagues [31] showed that mouse splenocytes treated with fucoidan, a polysaccharide from the brown alga *Fucus vesiculosus*, increased ROS production and efficiently resolved *L. donovani* infection. The anti-amastigote activity of ATA and AAE could be explained at least in part because of the capacity of these molecules to stimulate ROS production in macrophages that are infected or not with *Leishmania amazonensis*.

Together, our present findings show that *Stypopodium zonale* is an interesting source of natural products for drug discovery and the development of novel anti-protozoal agents. Atomaric acid and its methyl ester derivative, which exhibit leishmanicidal activity in vitro, may represent an attractive and safe candidate source for the development of drugs for the treatment of cutaneous leishmaniasis.

## 4. Experimental Section

### 4.1. Seaweed Sampling

The brown alga *Stypopodium zonale* was collected by the Instituto Brasileiro do Meio Ambiente e dos Recursos Naturais Renováveis (IBAMA), license number 45755 of 29/12/2014, by snorkeling at a depth of 2–3 m at Praia do Forno, Município de Búzios, Rio de Janeiro State (22°45′ S, 41°52′ W), Brazil, in February 2007; alga were identified by Dr. Lísia M. Gestinari (NUPEM-UFRJ Campus Macaé). A voucher specimen was deposited at the herbarium of the Universidade Federal do Rio de Janeiro (RFA 3823). Algae were washed in seawater to eliminate associated organisms and then were air-dried.

### 4.2. Extract Preparation and Procedures for Obtaining Meroditerpene

Air-dried and powdered algal material (130.0 g dry weight) was successively extracted with dichloromethane (2 L × 3 times, at room temperature for three weeks). Solvent was removed by vacuum yielding 11.8 g lipophilic extract (SZE). The chemical profile of the major compounds in SZE was determined by ^1^H NMR (nuclear magnetic resonance, 300 MHz) spectroscopy in a Bruker Advance spectrometer. SZE (2.5 g) was chromatographed on a SiO_2_ flash column using an *n*-hexane-ethyl acetate (EtOAc) and methanol (MeOH) step gradient system; 20 fractions (F1–F20) were obtained. All fractions, i.e., F1–F20, were analyzed by thin layer chromatography using Kieselgel 60 F_254_ aluminum support plates (Merck, Rio de Janeiro, RJ, Brazil). Fraction F4 (0.152 g), which contained the major compound in SZE, was eluted with 20% EtOAc in *n*-hexane, re-chromatographed on a SiO_2_ column with 25% EtOAc in *n*-hexane and, finally, 0.0036 g of the meroditerpenoid atomaric acid (ATA) was isolated.

### 4.3. Preparation of the Methyl Ester of Atomaric Acid (AAE)

To evaluate possible structural modifications on *S. zonale* compounds for effects on anti-leishmanial activity, the methyl ester of atomaric acid (AAE) was obtained after a methylation reaction of the extract. Briefly, 1.0 g of SZE was dissolved in a mixture of CHCl_3_–MeOH (4:1) and fresh diazomethane (CH_2_N_2_) in an excess of ethyl ether solution. After overnight magnetic stirring of the mixture, it was fractionated by silica gel vacuum liquid chromatography and eluted with increasing amounts of EtOAc in *n*-hexane. From the 12 fractions (F1–F12) that were obtained, F2 (0.2470 g) was chromatographed in a silica gel column and eluted with 15% EtOAc in *n*-hexane to yield 0.1360 g purified AAE.

### 4.4. Structural Elucidation

Chemical structures of the purified compounds were established by a comparison of previously reported ^1^H NMR, ^13^C NMR, mass spectrometry, and infrared spectroscopy data [22,23], and meroditerpenes, atomaric acid (ATA), and its methyl ester derivative (AAE) were identified (Figure 1).

### 4.5. Ethics Statement

All animal experiments were performed in strict accordance with the Brazilian animal protection law (Lei Arouca number 11.794/08) of the National Council for the Control of Animal Experimentation (CONCEA, Rio de Janeiro, Brazil). The study protocol was approved by the Committee for Animal Use of the Universidade Federal do Rio de Janeiro (CEUA Permit Number: 128/15; CONCEA Protocol: 01200.001568/2013-87).

### 4.6. Parasite Culture

*L. amazonensis* (WHOM/BR/75/Josefa) promastigotes were cultured at 26 °C in Schneider’s insect medium (Sigma, St. Louis, MO, USA), 10% fetal calf serum (FCS, Gibco, Frederick, MD, USA), and 20 μg/mL gentamycin (Schering-Plough, Rio de Janeiro, RJ, Brazil).

### 4.7. Anti-Promastigote Activity

The leishmanicidal properties of SZE, ATA, and AAE were evaluated by measuring promastigote viability. Stationary-phase promastigotes were treated with different concentrations of SZE, ATA, or AAE, and parasite survival was estimated by counting viable/motile forms in a hematocytometer during five days of culture at 26 °C. Data are expressed as the number of live parasites. As controls, promastigotes were maintained in culture medium and treated with the vehicle, dimethyl sulfoxide (DMSO; Sigma, St. Louis, MO, USA).

### 4.8. Anti-Amastigote Activity

Mouse peritoneal macrophages obtained after stimulation with 3% thioglycolate for three days were harvested in RPMI 1640 medium (LGC Biotec, Cotia, SP, Brazil) and cultured in 24-well plates for 2 h until they were adherent at 35 °C, 5% CO_2_. Non-adherent cells were removed, and macrophages were incubated overnight, as above, in RPMI with 10% FCS. Adherent macrophages were infected with *L. amazonensis* promastigotes (stationary growth phase) at a 10:1 parasite/macrophage ratio for 1 h at 35 °C, 5% CO_2_. Free parasites were washed out with 0.01 M phosphate buffered saline (PBS), and cultures were maintained as above for 24 h at 35 °C, 5% CO_2_. Infected macrophage cultures were treated with different concentrations of SZE, ATA, or AAE for an additional 24 h at 35 °C, 5% CO_2_. The cultures were then washed in PBS, fixed and stained with Giemsa. The number of amastigotes per macrophage and the percentage of infected macrophages were determined by counting at least 200 cells in triplicate cultures. Infectivity index was obtained by multiplying the percentage of infected macrophages by the mean number of amastigotes per infected macrophage. Amphotericin B used as a positive control was from Cristália (Itapira, SP, Brazil). The results were expressed as the percentage of killing compared with untreated controls.

### 4.9. Cytotoxicity for Host Macrophages

Mouse peritoneal macrophages that adhered to 96-well plates were treated with different concentrations of SZE, ATA or AAE for 24 h. Cell viability was determined using 1 mg/mL XTT (2,3-bis(2-methoxy-4-nitro-5-sulfophenyl)-2*H*-tetrazolium-5-carboxinilide inner salt, Sigma) with 200 μM PMS (Phenazine methosulfate, Sigma). After 3 h incubation, the reaction product was quantified at 450 nm. Data are expressed as the percentage of viable cells compared with untreated controls [32].

### 4.10. Nitric Oxide Production

Thioglycolate-stimulated peritoneal macrophages were obtained as described above (at 10^6^ cells/well in 24-well plates) and were either activated with 200 ng/mL IFN-γ (eBioscience, San Diego, CA, USA) or left untreated. After incubation for 24 h at 35 °C, 5% CO_2_, cells were treated with 100 μM ATA or AAE. Nitrite concentrations in culture supernatants were determined using the Griess method. The reaction was read at 540 nm, and the concentration of NO_2_^−^ was determined based on a standard curve of sodium nitrite. Data were expressed as the micromolar concentrations of nitrite [33].

### 4.11. Detection of Reactive Oxygen Species (ROS)

Mouse peritoneal macrophages that adhered to 96-well opaque culture plates were infected or not with *L. amazonensis* promastigotes. At 24 h post-infection, cultures were treated with 100 μM ATA or AAE and stimulated or not with 1 μg/mL phorbol 12-myristate13-acetate (PMA, Sigma). Cells were stained with 50 μM dihydrorhodamine 123 (DHR 123, Life Technologies, Waltham, MA, USA), and ROS was measured immediately using 500/526 nm excitation/emission wavelengths.

## Figures and Tables

**Table 1:** In vitro leishmanicidal effect and cytotoxicity results for ATA and AAE.

Compound	CC_50_	IC_50_	SI
ATA	169.5 μM (75 μg/mL)	20.2 μM (9 μg/mL)	8.4 (8.3)
AAE	262.5 μM (209 μg/mL)	22.9 μM (10 μg/mL)	11.5 (21)

The selectivity index (SI) is defined as the ratio of CC_50_ on murine peritoneal macrophages to IC_50_ on *L. amazonensis* intracellular amastigotes.
